# mSphere of Influence: Autoantibodies during malaria—a historical observation taking the modern spotlight

**DOI:** 10.1128/msphere.00906-25

**Published:** 2026-09-22

**Authors:** Juan Rivera-Correa

**Affiliations:** 1 Biological Sciences Department, New York City College of Technology, City University of New York 14780, Brooklyn, New York, USA; 2 Biology PhD Program, CUNY Graduate Center 14772, New York, New York, USA; Virginia-Maryland College of Veterinary Medicine, Blacksburg, Virginia, USA

**Keywords:** malaria, *Plasmodium*, autoimmunity, antibodies

## Abstract

Juan Rivera-Correa works in the field of immunology of tropical infectious diseases, focusing on the role of autoimmune responses in malaria. In this mSphere of Influence article, he discusses the papers by Rosenberg and Daniel-Ribeiro, which were two of the first papers to report the presence of autoantibodies in malaria patients, changing the paradigm and blurring the line between autoimmune disorders and infectious diseases.

## COMMENTARY

Many infectious diseases lead to pathology in the infected host. Although pathogen-induced damage has traditionally been regarded as the major pathology driver in the host, it has become increasingly recognized that host immune and autoimmune responses are major contributors to infection-induced pathology during relevant human infections such as malaria, HIV, tuberculosis, and COVID-19 (1, 2). Malaria caused by *Plasmodium* protozoan parasites is one of the deadliest and most prevalent global infections (3). In the past decade, pathological autoantibodies (autoimmune antibodies) have been described as major promoters of various malaria-associated pathologies, including anemia, cerebral malaria, and renal damage (4, 5). Curiously, two of the first reports of autoantibodies date back to the early 1970s (6) and the 1980s (7). Decades later, these observational findings were thoroughly explored and linked with pathological mechanisms in both patients and mouse models. This work led to what became the basis of my research interests in how autoantibodies can directly promote pathology by targeting self-antigens in host cells, promoting malaria anemia, and other pathologies during infection (8).

## WHAT THEY DISCOVERED

In reference 6, they present the first evidence of autoimmunity during malaria. They measured IgM antibodies that reacted against human red blood cell antigens in anemic malaria patients. Almost 10 years later (7), they performed a more robust approach, analyzing 14 autoantibodies in individuals from a malaria-endemic region, using indirect immunofluorescence and/or passive hemagglutination techniques. This work described anti-nuclear antibodies as the autoantibodies present in these samples, representing 87% of all total autoantibodies screened. These studies were limited to reporting the presence of these autoantibodies without thoroughly exploring the mechanism and clinical implications due to the limited technology at the time. Altogether, these studies established the presence of autoantibodies during human malaria, becoming classical reports linking human infectious diseases with autoimmunity.

## HOW THEY CHANGED ME

More than 30 years after these findings, I worked on my thesis characterizing the role of autoantibodies in promoting malaria anemia in mouse models and patients. At the time, our findings sounded novel to my basic immunology and infectious disease knowledge, where autoimmunity and infectious disease seem like exclusionary pathological scenarios, and I did not think there was an overlap. After finding these classic papers reporting these observations, my scientific reasoning became more inclusive, and I realized that immune scenarios are never isolated but instead could be occurring all at the same time, influencing each other and contributing to the highly variable clinical outcomes that we see in humans. This fueled my interest in finding mechanisms that could explain the role of autoantibodies during malaria and has led to the major research questions in my laboratory. Following these historical observations, our work first identified high levels of anti-red blood cell antibodies in *Plasmodium yoelii* malaria-infected mice. After a series of mechanistic *in vitro* and *in vivo* studies, we identified the cell membrane phospholipid phosphatidylserine (PS) as one of the main specific red blood cell antigens being targeted in both mice and *Plasmodium falciparum* and *Plasmodium vivax* malaria patients (9–13). After our studies, anti-PS and other autoantibodies have been reported by other groups in different malaria cohorts around the world (14–18), showing their great significance and potential as clinical biomarkers of disease. Additionally, we hypothesized that the clinical significance of these autoantibodies could be expanded to other highly inflammatory infections such as other parasitic and viral infections. Accordingly, we have found increased levels of anti-PS autoantibodies in different highly inflammatory infections such as Zika virus (19, 20), African trypanosomiasis (21), and dirofilariasis (22). Curiously, autoantibodies have gotten the modern spotlight due to their presence and pathological correlation in the COVID-19 pandemic (23–26), where autoantibodies were found to be major drivers of pathologies such as Long-COVID (27). Altogether, these classical findings have shaped my research program studying the presence and roles of autoantibodies during infectious diseases.

## WHERE DO WE GO NEXT

Autoimmune-induced pathology during infections has recently been spotlighted due to the growing number of high-profile infections that report the presence of different pathological autoantibodies, such as COVID-19. These infection fields are utilizing the foundational work that has been laid out for malaria and other infections and expanding it to contemporary infections. In malaria, other groups have reported data that challenge the norm and found evidence that autoantibodies could even have beneficial roles (28), adding to the complexity of this phenomenon. The recent high-throughput antibody screening technologies, such as autoantibody microarrays (29) and systems serology (30), will be useful tools to understand both the presence and roles that autoantibodies play during infectious diseases by assessing hundreds of antigens and functions simultaneously. Additionally, to further dissect the mechanism leading to these phenomena, the high-throughput analysis of global and antigen-specific B-cell populations is needed through cutting-edge technologies such as single-cell sequencing with V(D)J profiling, B-cell receptor sequencing, and mass cytometry (31). This field will likely keep growing due to these high-throughput B-cell and antibody technologies, which will clarify and establish the clinical and biomarker potential of autoantibodies during infectious diseases. Lastly, the changing understanding and acceptance of the pathological role of autoantibodies during infectious diseases could shift our perspective on how we treat them. Clinicians will have to evaluate the possibility of combining anti-infective treatments with antibody immunomodulators to effectively treat severe complications in infectious disease patients. Collectively, these historical observations of autoantibodies during malaria have taken on modern relevance and a spotlight role in understanding human infectious disease pathogenesis.
